# Immune-mediated enterocolitis is associated with immune checkpoint inhibitors: A pharmacovigilance study from the FDA Adverse Event Reporting System (FAERS) database

**DOI:** 10.1371/journal.pone.0325760

**Published:** 2025-06-04

**Authors:** Connor Frey

**Affiliations:** Department of Medicine, University of British Columbia, Vancouver, British Columbia, Canada; Nihon University School of Medicine, JAPAN

## Abstract

**Purpose:**

Immune checkpoint inhibitors (ICIs) have revolutionized cancer treatment by demonstrating significant efficacy across multiple malignancies. However, by interfering with immune regulatory pathways, they can lead to immune-related adverse events (irAEs), including immune-mediated enterocolitis. This study aimed to evaluate the real-world risk of immune-mediated enterocolitis across different ICIs using data from the FDA’s Adverse Event Reporting System (FAERS).

**Methods:**

A disproportionality analysis was conducted using FAERS data to assess the association between different ICIs and the risk of immune-mediated enterocolitis. The risk was analyzed across three ICI classes: cytotoxic T-lymphocyte-associated protein 4 (CTLA-4) inhibitors, programmed death-1 (PD-1) inhibitors, and programmed death-ligand 1 (PD-L1) inhibitors.

**Results:**

The analysis revealed significant variability in the risk of immune-mediated enterocolitis among ICIs. CTLA-4 inhibitors, particularly tremelimumab and ipilimumab, exhibited the strongest association with enterocolitis. Among PD-1 inhibitors, nivolumab demonstrated the highest risk, while PD-L1 inhibitors, including durvalumab and atezolizumab, had a lower but still notable association.

**Conclusions:**

These findings underscore the need for vigilant monitoring and early intervention in patients receiving ICIs. The differential risk profile among ICIs suggests that physicians should consider enterocolitis risk when selecting and managing immunotherapy regimens.

## Introduction

The advent of immune checkpoint inhibitors (ICI) has transformed cancer treatment, fundamentally changing oncologists’ approach to various malignancies [1]. These therapies, targeting PD-1, PD-L1, and CTLA-4, inhibit regulatory pathways that typically downregulate the immune system [2]. By blocking these checkpoints, ICIs allow CD8^+^ T-cells to more effectively detect and eliminate cancer cells that would otherwise evade immune surveillance [3]. Despite the benefits of ICIs, the interruption of immune checkpoints can inadvertently lead to a spectrum of immune-related adverse events (irAEs) [4]. Among these, immune-mediated enterocolitis (IME) has emerged as a particularly noteworthy and potentially serious complication [5]. IME involves inflammation of the gastrointestinal tract, specifically the small and/or large intestine, and presents a clinical challenge due to its varied manifestations.

This study provides an updated assessment of the real-world risk of IME with ICIs. By analyzing current data, it serves as a resource for oncologists and gastroenterologists, particularly in gastrointestinal cancers, where distinguishing between disease symptoms and ICI-induced effects is challenging. The study aims to raise awareness of IME and contribute to optimizing treatment strategies, ultimately improving patient outcomes and quality of life.

## Methods

The methods have been described previously [6,7]. FAERS serves as a comprehensive repository for documented adverse drug reactions (ADRs) submitted by healthcare professionals, manufacturers, and patients, playing a crucial role in post-market drug safety monitoring. The system enables the identification of potential safety concerns through disproportionality analyses, a statistical method that compares the incidence of a specific adverse event (AE) associated with a drug to the background incidence of the same AE for all other pharmaceuticals in the database. This approach helps determine whether a particular drug is linked to a higher-than-expected frequency of an AE, thereby facilitating early detection of potential safety risks.

For this study, OpenVigil 2.1 (OpenVigil, Kiel, Germany), a data-mining tool designed to facilitate FAERS data analysis, was employed. OpenVigil allows for the execution of customized queries to extract relevant data efficiently. Specific queries were formulated to retrieve data corresponding to the Medical Dictionary for Regulatory Activities (MedDRA) Lower Level Term (LLT) “immune-mediated enterocolitis.” Data associated with each immune checkpoint inhibitor was systematically extracted for further evaluation. At no point did the author have access to information which would identify a patient in the database, all data were fully anonymized. As only publicly available patient data were used in this study, no ethics or IRB approval was required.

The analysis included adverse event reports submitted to FAERS from the fourth quarter of 2003 through the first quarter of 2024, allowing for a comprehensive assessment of long-term trends in reporting patterns. To evaluate the association between immune checkpoint inhibitors and immune-mediated enterocolitis, several statistical measures were applied, including the Proportional Reporting Ratio (PRR), Chi-squared (χ2) test, Reported Relative Risk (RRR), and Reporting Odds Ratios (RORs). A PRR greater than 2.00 suggests that the AE is reported at least twice as frequently for the drug in question compared to other drugs. A χ2 value exceeding 4.00 indicates statistical significance. The RRR quantifies the relative risk of experiencing the AE when taking the drug compared to other drugs, with values greater than 2.00 suggesting at least twice the risk. The ROR assesses the likelihood of the AE being reported for the drug relative to other pharmaceuticals, with values greater than 1.00 indicating an increased likelihood of association. These statistical measures collectively provide a robust framework for evaluating potential safety concerns associated with immune checkpoint inhibitors.

## Results

### PD-1 Inhibitors

Nivolumab exhibited the highest association with IME among PD-1 inhibitors, with a PRR of 310.991 and a χ2 of 89,402.540 (Table 1). The RRR was calculated at 223.535, and the ROR was 319.047 (95% Confidence Interval, CI: 284.058, 358.347). There were 404 reports of IME associated with nivolumab, accounting for 39.30% of the total reports of adverse events for the drug. Pembrolizumab also showed a considerable association with IME, though to a lesser extent than nivolumab. The PRR was 112.798, with a χ2 of 25,407.797. The RRR was 90.314, and the ROR was 113.955 (95% CI: 100.088, 129.742). There were 288 reports of IME, making up 25.18% of the total reports for pembrolizumab. In contrast, cemiplimab, dostarlimab, and tislelizumab demonstrated much lower associations with IME. Cemiplimab had a PRR of 63.593 and an ROR of 64.046 (95% CI: 28.642, 143.212) with 6 reports of IME, which constituted only 0.42% of the total reports. Dostarlimab and tislelizumab each had only 1 report of IME, with both contributing to 0.07% of the total reports for these drugs. Their RORs were 19.840 (95% CI: 2.787, 141.260) and 8.681 (95% CI: 1.221, 61.731), respectively.

**Table 1:** Statistical Data and Number of Reports for Immune-Mediated Enterocolitis Associated with the PD-1, PD-L1, and CTLA-4 Immune Checkpoint Inhibitors.

Drug	PRR	χ2	RRR	ROR (95% CI)	Reports of IME	Total AEs for the Drug	Percentage of Total Reports for IME
**PD-1**							
Nivolumab	310.991	89402.540	223.535	319.047 (284.058, 358.347)	404	1028	39.30%
Pembrolizumab	112.798	25407.797	90.314	113.955 (100.088, 129.742)	288	1144	25.18%
Cemiplimab	63.593	308.443	63.331	64.046 (28.642, 143.212)	6	1426	0.42%
Dostarlimab	19.798	3.997	19.785	19.840 (2.787, 141.260)	1	1431	0.07%
Tislelizumab	8.673	1.283	8.668	8.681 (1.221, 61.731)	1	1431	0.07%
**PD-L1**							
Durvalumab	90.853	3953.527	87.904	91.758 (68.514, 122.887)	47	1385	3.39%
Atezolizumab	39.017	1298.516	38.035	39.182 (28.249, 54.346)	37	1395	2.65%
Avelumab	9.459	7.842	9.448	9.469 (2.364, 37.925)	2	1430	0.14%
**CTLA-4**							
Tremelimumab	604.138	19977.830	589.396	647.307 (457.548, 915.764)	35	1397	2.51%
Ipilimumab	273.460	53649.266	228.176	280.692 (243.859, 323.088)	238	1194	19.93%

PRR, Proportional Reporting Ratio; χ^2^, Chi-Squared; RRR, Reported Relative Risk; ROR, Reporting Odds Ratio; CI, Confidence Interval; IME, Immune-Mediated Enterocolitis; AE, Adverse Event.

### PD-L1 Inhibitors

Durvalumab showed the highest association with IME among PD-L1 inhibitors, with a PRR of 90.853 and a χ2 of 3,953.527 (Table 1). The RRR was 87.904, and the ROR was 91.758 (95% CI: 68.514, 122.887). A total of 47 reports of IME were associated with durvalumab, representing 3.39% of the total reports for the drug. Atezolizumab was the second most associated PD-L1 inhibitor with IME, having a PRR of 39.017 and an ROR of 39.182 (95% CI: 28.249, 54.346). There were 37 reports of IME, accounting for 2.65% of the total reports for atezolizumab. Avelumab showed the lowest association within this group, with a PRR of 9.459 and an ROR of 9.469 (95% CI: 2.364, 37.925). Only 2 reports of IME were linked to avelumab, representing 0.14% of the total reports.

### CTLA-4 Inhibitors

Among CTLA-4 inhibitors and all the immune checkpoint inhibitors, tremelimumab exhibited the highest association with IME, with a PRR of 604.138 and a χ2 of 19,977.830 (Table 1). The RRR was calculated at 589.396, and the ROR was 647.307 (95% CI: 457.548, 915.764). There were 35 reports of IME associated with tremelimumab, accounting for 2.51% of the total reports for this drug. Lastly, ipilimumab also showed a strong association with IME, with a PRR of 273.460 and a χ2 of 53,649.266. The RRR was 228.176, and the ROR was 280.692 (95% CI: 243.859, 323.088). A total of 238 reports of IME were associated with ipilimumab, representing 19.93% of the total reports

## Discussion

The results demonstrate that the risk of IME varies considerably across different classes of ICIs, with CTLA-4 inhibitors showing the strongest associations. Tremelimumab had the highest PRR and ROR. This finding is consistent with previous studies that have reported higher rates of gastrointestinal toxicities with CTLA-4 inhibitors and is higher when PD-1/CTLA-4 agents are combined [8,9]. This is possibly due to the proposed mechanism wherein PD-1/PD-L1 blockade occurs during late T-cell proliferation and, thus, causes a localized immune reaction, whereas CTLA-4 blockade induces CD4^+^ T-cell activation, resulting in a generalized immune response [10]. Among PD-1 inhibitors, nivolumab and pembrolizumab were associated with a significantly higher risk of IME, with nivolumab exhibiting the highest number of reported cases. On the other hand, the PD-L1 inhibitors, particularly durvalumab and atezolizumab, showed a lower but still notable association with IME. Based on prior studies, the incidence of colitis of any grade ranged from 5.7% to 39.1% for CTLA-4 inhibitors and from 0.7% to 31.6% for PD-1/PD-L1 inhibitors [10–12].

The heightened risk of IME associated with certain ICIs underscores the importance of vigilant monitoring and early intervention. For oncologists and gastroenterologists, understanding the specific risks associated with each ICI can inform treatment decisions, particularly in patients who may be predisposed to gastrointestinal complications or those with pre-existing conditions [13]. The findings suggest that healthcare providers should maintain a high index of suspicion for IME in patients receiving ICI, especially tremelimumab or ipilimumab, given the significantly elevated risk with these agents [14,15]. Early recognition and management of IME are crucial to preventing severe complications [16].

The data presented in this study also opens avenues for further research. Prospective studies are needed to better understand the underlying mechanisms that contribute to the varying risks of IME across different ICIs. Additionally, research into biomarkers that could predict susceptibility to IME would be valuable in personalizing ICI therapy and mitigating the risk of severe adverse events. Moreover, the study underscores the importance of real-world data in complementing clinical trial findings. The use of FAERS to analyse AE reports provides a broader picture of the risks associated with ICIs in diverse patient populations and settings. However, the reliance on spontaneous reporting systems also introduces potential biases, such as underreporting or misclassification, which should be considered when interpreting the findings.

This study provides a comprehensive analysis of the incidence and risk of immune-mediated enterocolitis associated with immune checkpoint inhibitors. The findings underscore the significant variability in risk across different ICIs, with CTLA-4 inhibitors posing the highest risk, in agreeance with previous literature. These insights are crucial for optimizing the management of patients undergoing ICI therapy and highlight the need for continued research into the mechanisms and predictors of ICI-related adverse events. By improving our understanding of IME and its association with different ICIs, healthcare providers can better balance the benefits of immunotherapy with the potential risks, ultimately leading to improved patient outcomes.

**Five key points**

- The study highlights varying risks of immune-mediated enterocolitis across immune checkpoint inhibitors, aiding physicians in treatment selection and patient monitoring.
- Among CTLA-4 inhibitors, tremelimumab shows the strongest association with IME.
- Nivolumab carries the highest IME risk among PD-1 inhibitors, while PD-L1 inhibitors have lower but notable risks.
- Understanding IME risk helps balance treatment efficacy and safety, guiding immunotherapy use.
- Vigilant monitoring is essential to prevent severe complications in ICI-treated patients.
